# Genomic analysis and clinical management of adolescent cutaneous melanoma

**DOI:** 10.1111/pcmr.12574

**Published:** 2017-04-19

**Authors:** Roy Rabbie, Mamunur Rashid, Ana M. Arance, Marcelo Sánchez, Gemma Tell‐Marti, Miriam Potrony, Carles Conill, Remco van Doorn, Stefan Dentro, Nelleke A. Gruis, Pippa Corrie, Vivek Iyer, Carla Daniela Robles‐Espinoza, Joan A. Puig‐Butille, Susana Puig, David J. Adams

**Affiliations:** ^1^ Experimental Cancer GeneticsThe Wellcome Trust Sanger InstituteHinxtonCambridgeshireUK; ^2^ Department of OncologyCambridge University Hospitals National Health Service Foundation TrustCambridgeUK; ^3^ Department of Medical Oncology and Targeted Therapeutics in Solid Tumors Group (IDIBAPS)Hospital Clınic de BarcelonaBarcelonaSpain; ^4^ Melanoma UnitRadiology ServiceHospital ClınicIDIBAPSUniversity of BarcelonaBarcelonaSpain; ^5^ Melanoma UnitDepartment of DermatologyHospital Clınic de BarcelonaBarcelonaSpain; ^6^ Centre of Biomedical Research on Rare Diseases (CIBERER)ISCIIIBarcelonaSpain; ^7^ Melanoma UnitDepartment of DermatologyHospital Clınic de BarcelonaIDIBAPSBarcelona UniversityBarcelonaSpain; ^8^ Melanoma UnitRadiotherapy OncologyHospital ClınicIDIBAPSBarcelona UniversityBarcelonaSpain; ^9^ Leiden University Medical CentreLeidenThe Netherlands; ^10^ Laboratorio Internacional de Investigacion sobre el Genoma HumanoUniversidad Nacional Autonoma de MexicoSantiago de QueretaroMexico; ^11^ Biochemistry and Molecular Genetics DepartmentMelanoma UnitHospital Clinic de BarcelonaIDIBAPSBarcelonaSpain

**Keywords:** adolescent melanoma, germline mutation, ultraviolet radiation, BRAF mutation, immunotherapy

## Abstract

Melanoma in young children is rare; however, its incidence in adolescents and young adults is rising. We describe the clinical course of a 15‐year‐old female diagnosed with AJCC stage IB non‐ulcerated primary melanoma, who died from metastatic disease 4 years after diagnosis despite three lines of modern systemic therapy. We also present the complete genomic profile of her tumour and compare this to a further series of 13 adolescent melanomas and 275 adult cutaneous melanomas. A somatic *BRAF*^*V*^ ^*600E*^ mutation and a high mutational load equivalent to that found in adult melanoma and composed primarily of C>T mutations were observed. A germline genomic analysis alongside a series of 23 children and adolescents with melanoma revealed no mutations in known germline melanoma‐predisposing genes. Adolescent melanomas appear to have genomes that are as complex as those arising in adulthood and their clinical course can, as with adults, be unpredictable.

**Significance**

The survival from advanced melanoma in adults has been revolutionized by the introduction of immune checkpoint inhibitors and molecular targeted therapies. However, children and adolescents younger than 18 years have had limited access to the registration clinical trials. We present a detailed genomic analysis of a series of adolescent melanomas and the clinical course of one such patient who died from metastatic disease. A high mutational load was observed and suggests that immune‐based therapies may be relevant, but response cannot be guaranteed. Germline mutations in established adult melanoma‐predisposing genes were not evident in an extended childhood and adolescent series. Given the complexities around diagnosis and the paucity of prospective clinical studies for younger individuals, melanoma in this age group represents a particular clinical challenge requiring specialist management by a dedicated multidisciplinary team.

## Introduction

Melanoma in children is rare accounting for only 2% of all malignancies in patients younger than 20 years (Howlader et al., 2016). Melanoma in infancy and early childhood (1–10 years) comprises around 8% of newly diagnosed cases in young people, whereas adolescents (11–20 years) account for the majority (92%) of melanoma cases (Lorimer et al., 2016). Importantly, the incidence of melanoma in the adolescent population is rising at a rate of 2% per year (Austin et al., 2013). Melanocytic lesions in children comprise a heterogeneous group of neoplasms that can be broadly divided based on histology and age onset, and three major subtypes have been described (Barnhill and Kerl, 2006). Firstly, melanoma can arise in association with a pre‐existing, usually large, congenital melanocytic naevus (CMN) (Guegan et al., 2016; Trozak et al., 1975). The lifetime risk of malignant transformation from a CMN is 5–10% and 50% of these transformations are said to occur in the first decade of life (Bett, 2005; Krengel et al., 2006). The second type are termed spitzoid melanocytic tumours, which comprise a wider spectrum of histological variants including spitzoid melanoma and atypical Spitz tumours. The vast majority of Spitz naevi occur in individuals younger than 20 years and often arise on the extremities and face (Reed et al., 2013). The third subtype, generally occurring in adolescents, has been termed ‘conventional’ melanoma, owing to its shared clinical and histological features typical of adult melanoma. In contrast to infantile and childhood cases, post‐pubertal melanoma is most often sporadic, occurring as a de‐novo lesion in patients with fair‐coloured skin and substantial sun exposure (Wood, 2016).

Cutaneous melanoma in adults is characterised by a high prevalence of somatic mutations and the mutational pattern depicts a characteristic ultraviolet‐light (UV)‐induced signature associated with frequent transitions at dipyrimidine sites (Cancer Genome Atlas Network, 2015). A recent comprehensive genomic analysis found that melanomas from adolescents bear a remarkably similar mutational rate and spectrum to tumours from adults, suggesting that sun protection practices are important in early life (Anderson et al., 2009; Lu et al., 2015). In addition to its rarity and the low clinical suspicion for malignancy, there is recognition that melanomas in young people are commonly amelanotic and the clinico‐pathologic features may overlap with proliferative nodules and other benign skin lesions that are generally more common in children than adults (Cordoro et al., 2013; Moscarella et al., 2012). This can lead to delays both in diagnosis and treatment (Neier et al., 2012).

Several high‐risk mutations have been identified in melanoma‐dense families, including mutations in the cyclin‐dependent kinase inhibitor 2A (*CDKN2A*) gene (Cannon‐Albright et al., 1992)*,* the cyclin‐dependent kinase 4 (*CDK4*) gene (Zuo et al., 1996) and more recently in the Breast cancer 1 (*BRCA1*)‐associated protein 1 (*BAP1*) (Aoude et al., 2013; Wiesner et al., 2011) and protection of telomeres 1 (*POT1*) genes (Robles‐Espinoza et al., 2014; Shi et al., 2014). However, the prevalence of these predisposing mutations amongst younger patients is largely unknown. A deeper understanding of the molecular drivers of childhood and adolescent melanoma would advance our understanding of its pathogenesis, particularly the role of gene–environment interactions in susceptible cases and could help define particular high‐risk subgroups that might benefit from specialist screening and surveillance.

## Results

In this study, we present a detailed clinical history of one patient and an extensive genomic analysis of their germline and tumour and that of a wider series of adolescent and childhood melanomas.

### Patient presentation

**Figure 1:**
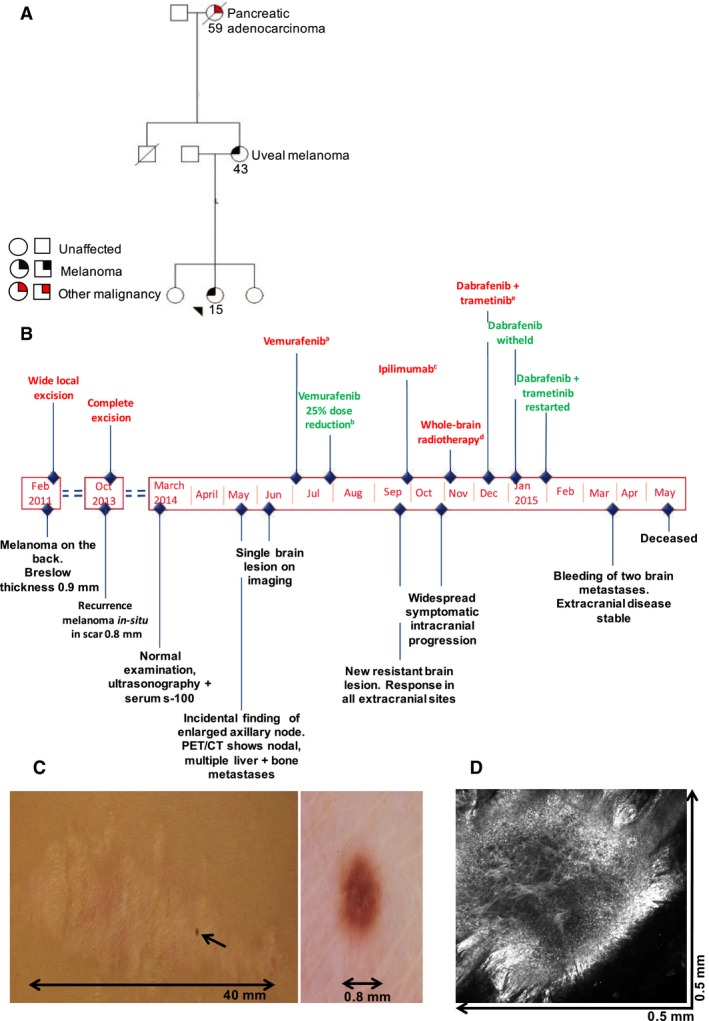
Clinical timeline of the 15‐year‐old index patient (M_4180). (A) Family pedigree. The proband is indicated with an arrow, ages at diagnosis are shown. (B) Timeline of diagnosis and treatment. (C) New pigmented melanoma in situ appearing in the centre of previous melanoma wide local excision scar. Accompanied dermoscopic image of the in‐situ melanoma prior to further wide local excision (beside). (D) Reflectance confocal microscopy at the dermo‐epidermal level, showing proliferation of dendritic atypical melanocytes. ^a^Vemurafenib starting dose 960 mg twice a day. ^b^Dose reduction vemurafenib to 720 mg twice a day. ^c^Ipilimumab 3 mg/kg every 3 weeks. ^d^Whole‐brain radiotherapy 10 Gy in 10 fractions. ^e^Dabrafenib 150 mg twice a day, trametinib 2 mg once a day.

**Figure 2:**
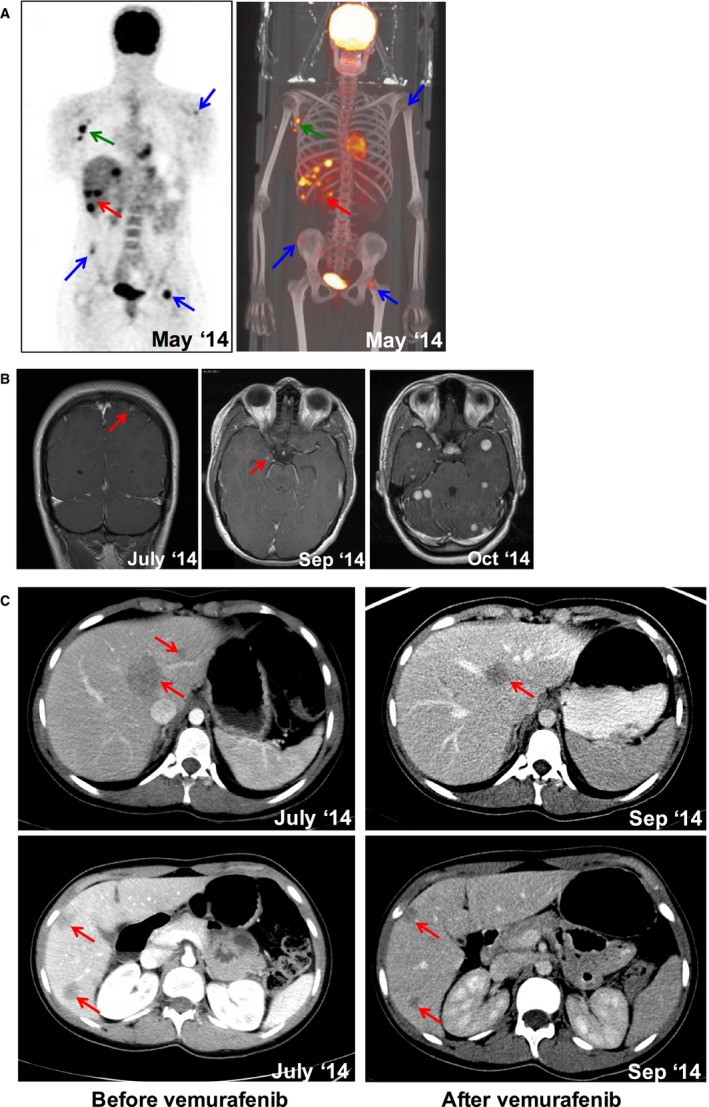
Radiological evaluation through treatment. (A) 18F‐FDG PET/CT alongside 3D colour reconstruction. Arrows indicate avid FDG tracer uptake in the right axilla, left humeral head, left femoral neck and right iliac crest (blue) as well as widespread liver uptake (red). (B) Post‐contrast T1‐weighted MR images showing tiny enhancing lesions in the left parietal lobe (July 2014) and right amygdala (September 2014). Axial post‐contrast MR images prior to whole‐brain radiotherapy showing multiple and supra‐ and infratentorial lesions with no significant mass effect (October 2014). (C) Cross‐sectional CT images of the liver post‐IV‐contrast in the portal phase. Baseline images show hypodense focal lesions corresponding to segment 1 in the left hepatic lobe (left upper) and the caudal segments of the right hepatic lobe (left lower). On the right, post‐treatment images indicate a partial response in all liver lesions (arrows).

The 15‐year‐old female described had blonde hair, blue eyes, skin phototype II on the Fitzpatrick Classification Scale (Fitzpatrick, 1988) and a history of multiple (>50) benign skin naevi. Her mother had a history of uveal melanoma, and her maternal grandmother had pancreatic adenocarcinoma (Figure 1A). She presented in February 2011 with an enlarging symmetric raised light brown papule on the right lower posterior chest wall at the level of the costal margin, which measured less than 1 cm in diameter. The lesion was removed by shave excision at her local hospital and was found to be a non‐ulcerated cutaneous malignant melanoma, Clark's level IV, Breslow thickness 0.9 mm and 6 mitoses/mm^2^ (Figure 1B). As the lesion extended to the excision margins, wide local excision was undertaken with subsequent clear margins. Ultrasonography revealed no pathological regional lymph nodes and she underwent active multimodality 6‐monthly surveillance. Two and a half years later in October 2013, a 0.8 mm pigmented lesion appeared in the centre of the existing wide local excision scar (Figure 1C). Dermoscopic examination revealed a homogeneous pattern and reflectance confocal microscopy showed atypical dendritic cells in the dermo‐epidermal junction (Figure 1D). This lesion was diagnosed as melanoma in situ, which was completely excised. In March 2014, no abnormalities were detected on surveillance clinical examination or ultrasonography and serum s‐100 levels were recorded as normal at 0.13 μg/l (normal <0.15 μg/l). However, 2 months later, during a separate clinic consultation for acne treatment, an enlarged lymph node was detected in the right axilla and serum s‐100 levels were now elevated to 0.7 μg/l. A PET/CT scan revealed avid FDG uptake in multiple liver and bone metastases as well as right axillary lymph nodes (Figure 2A). A single asymptomatic parietal lobe brain metastasis was also identified on imaging (Figure 2B). Three cutaneous metastases were evident, one of which was excised. Molecular analysis of the excised metastasis using PCR revealed a *BRAF* ^*V600E*^ mutation. In July 2014, she was commenced on systemic therapy with the BRAF kinase inhibitor, vemurafenib. Ten days into therapy, she experienced arthralgia, blepharitis, meibomian gland inflammation (presenting with suppuration from the sebaceous gland at the rim of the eyelids and treated with topical and oral antibiotics), as well as a widespread cutaneous rash necessitating interruption of treatment (Erfan et al., 2017; Figure S1). Treatment was reintroduced 2 weeks later at a 25% dose reduction. Repeat cross‐sectional imaging 2 months later showed a response in all the nodal and liver lesions (Figure 2C). There was also response in the parietal lobe lesion, but a new brain metastasis within the amygdala was now evident (Figure 2B). Vemurafenib was therefore stopped and, following a 3‐week washout, immune checkpoint inhibitor therapy with ipilimumab was commenced. Following the second cycle, she was admitted to hospital with migraine and unsteadiness of gait and neuroimaging revealed widespread multiple brain metastases (Figure 2B). Her symptoms improved with corticosteroids and whole‐brain radiotherapy. In December 2014, combination MAP kinase inhibitor therapy with dabrafenib and trametinib was commenced. Treatment was associated with pyrexia necessitating brief interruption of dabrafenib, but subsequent resumption of the combination regimen. At the end of March 2015, she was readmitted with a sudden‐onset severe headache. Imaging revealed bleeding and perilesional oedema into two existing brain metastasis and the appearance of a further new brain metastasis. She died from progressive metastatic melanoma 2 months later.

### Tumour genomic analyses

**Figure 3:**
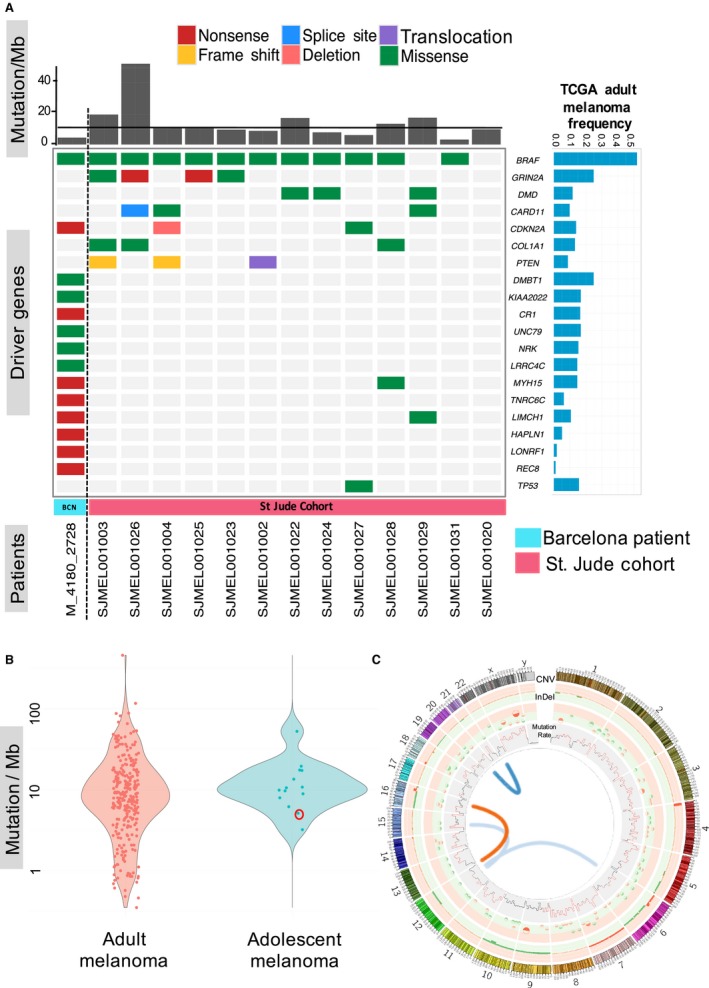
Somatic genomic analyses of adolescent melanoma. (A) Mutational landscape of adolescent melanoma. Driver mutations from the patient described are shown in the first column on the left‐hand side. Remaining cases are from Lu et al. (2015) and indicate the 13 conventional adolescent melanoma patients described within this cohort and for whom genome sequencing data was available. Bar chart across the top panel shows the mutation rate per megabase (Mb) while the right panel shows the mutational frequency in adult cutaneous melanoma found in The Cancer Genome Altas (TCGA; Cancer Genome Atlas Network, 2015), straight line indicates the median number of mutations across all patients. Genes were selected based on those most frequently mutated in The Cancer Genome Atlas (adult) and in Lu et al. (childhood and adolescent; Lu et al., 2015), as well as the loss‐of‐function mutations detected in this 15‐year‐old patient. A number of commonly mutated genes identified in the TCGA melanoma cohort are omitted owing to the absence of mutations of these genes in our adolescent data set (including *NRAS*,*NF1*,*MAP2K1* and *RB1*). (B) Cluster plot of mutational frequency of adolescent versus adult cutaneous melanoma. The index patient described is circled in red. (C) Circos plot of somatic changes in the 15‐year‐old patient described. The outermost track shows large copy number gains (red) and losses (green) (Table S8). Middle track shows small insertions and deletions (Table S9). The inner most track shows mutations per Mb (regions marked in red have mutation rates higher than 15 mutations/Mb). Interchromosomal translocations are shown in the centre and were seen in; t(12;6)(q21;q2), t(12;15)(q14;q1), t(16;12)(q23;q2) and t(20;22)(q13;q32) (Table S10).

Whole‐genome sequencing of a cutaneous metastasis and matched germline DNA from the patient described above revealed somatic mutations in melanoma driver genes including a *BRAF* ^*V600E*^ mutation, and a truncating *CDKN2A* mutation (Figure 3A). In total, we identified 133 mutations in the protein‐coding region of the genome, of which 89 were protein‐altering and 44 were silent (non‐synonymous to silent mutation ratio = 2.022; Tables S4 and S5). 15,853 somatic mutations were identified genome wide with a mutation frequency of 5.12 mutations per megabase (Figure 3A, C). The tumour displayed a disproportionately high level of cytidine to thymidine (C>T) transitions accounting for >80% of all nucleotide changes. The mutational spectrum bore closest resemblance to the UV‐exposure signature (signature 7) described by Alexandrov et al. (2013) (cosine similarity test 0.63; Figure S2). We validated 42 randomly selected loci via Sanger sequencing of tumour and germline DNA and found 36 (86%) to be true somatic variants (Table S7). A further 13 ‘conventional’ melanomas (so‐called due to their shared clinical and histological features typical of adult cutaneous melanoma) were identified from Lu et al. (2015). These patients had a median age of 16 years (13–20) and ranged from stage IB to IV disease at initial diagnosis. The primary tumours were generally from sun exposed sites (six from the head and neck, three from the trunk, three from the extremities and one unknown) and were mainly of common histological subtypes (six nodular, five superficial spreading, one acral and one unknown; Table S1). Pooling variants from our patient with somatic variant calls from these 13 conventional melanomas revealed a median of 10.23 mutations per megabase (3.21–52.65; Figure 3A; Table S6). We obtained further mutation data from 275 adult cutaneous melanomas from The Cancer Genome Atlas (mean age 56.62 years). A Wilcoxon test comparing these to the adolescent melanoma series did not reveal any significant difference between the mutation rates of adolescent vs adult cutaneous melanoma (P value = 0.2721).

### Germline genomic analyses

We investigated this 15‐year‐old patient's germline genome for known melanoma‐predisposing genes including *CDKN2A, CDK4* and *BAP1* but failed to find any rearrangements, copy number neutral changes, point mutations or other alternations that may explain her presentation. A wider analysis of 23 additional children and adolescents, including five new cases with resected primary melanoma that we whole‐genome‐sequenced for this study and 18 children described in Lu et al. (2015), also failed to identify variants in established melanoma‐predisposing genes. These five new cases had a median age of 10 years (6–16), were all of the superficial spreading histological subtype and had AJCC stage I disease at first presentation, while the remaining 18 cases identified from Lu et al. had a median age of 15 years (9 months–20 years) and included a wider spectrum of both stages and histological subtypes (Table S1). We noted that our patient carried R142H and V60L alleles in the melanocortin 1 receptor (*MC1R*) gene, contributing to her pale complexion (Garcia‐Borron et al., 2014) (Figure S1). Other *MC1R* variants were also discovered in the children and adolescents analysed in our study (Table S3). In view of the emerging evidence implicating telomere dysregulation in familial melanoma (Robles‐Espinoza et al., 2014; Shi et al., 2014), we further searched for alterations in genes encoding the shelterin protein complex that protect the ends of telomeres. In particular, the protection of telomeres 1 (*POT1*) gene, adrenocortical dysplasia homolog (*ACD*) gene and telomeric repeat binding factor 2 interacting protein (*TERF2IP*) genes have been shown to be important in some melanoma families (Aoude et al., 2015). We found 1 of 24 patients carried a missense mutation in *TERF2IP* (allele frequency 0.00378 in The Exome Aggregation Consortium (ExAC) (Table S3), although the pathogenicity of this mutation is unknown.

## Discussion

Metastatic spread of melanoma is relatively rare amongst children; however, there are data that suggests that when this occurs the prognosis is particularly poor (Strouse et al., 2005). The adolescent described in this study presented with a AJCC stage IB primary melanoma, which is associated with a 95% 5‐year survival (Balch et al., 2009). Despite this, she developed extensive metastases 3 years after diagnosis and died of metastatic disease within 12 months despite three lines of modern systemic therapies known to offer potential for survival gain.

Notably, and as reported previously (Lu et al., 2015), we identified a preponderance of UV‐induced mutations across ‘conventional’ adolescent melanomas, which was unexpected given the relatively limited exposure to UV light compared to an adult population. This 15‐year‐old patient had intermittent sun exposure amounting to approximately 120 h/yr, yet was always appropriately sun protected. We were unable to find germline predisposing alleles in an extended series of children and adolescents, suggesting that established high‐penetrance predisposition genes do not explain most cases. However, many of these patients carried *R* variants of *MC1R* associated with red hair, freckles and pale skin (Valverde et al., 1995).

Given the variability in clinical behaviour, wide histological variation and the rarity of melanoma in infancy and early childhood, studies in this population are scarce. Consequently, our understanding of the pathogenesis in this younger cohort is more limited. Analysis of a recent large national data set of over 350 000 melanoma patients showed that children (1–10 years) and adolescents (11–20 years) had differing survivals, suggesting inherent differences in the biology of the disease (Lorimer et al., 2016). The distinct clinical and histopathological features of melanomas arising in a CMN and Spitzoid tumours suggest that their molecular features are likely to be very different from the ‘conventional’ adolescent tumours described herein (Kinsler et al., 2013; Lu et al., 2015; Shakhova et al., 2012). Additional studies on the genomic evolution of these rarer subtypes could help facilitate improved diagnostics and tailored therapies.

The reason for the rise in incidence of melanoma during adolescence remains unclear; however, the finding of a high mutational load driven by UV exposure supports the need for education and behavioural modification as an important preventative strategy starting in early life (Green et al., 2011). The strong therapeutic effect of immune checkpoint blockade in some patients has been linked to the expression of neoantigens, mutant peptides presented by MHC Class I. A higher overall mutational burden would be expected to lead to the expression of more neoantigens, with mutation number being associated with improved efficacy of immunotherapy (Snyder et al., 2014; Van Allen et al., 2015). This adolescent developed metastatic disease at 18 years and accessed a range of modern treatments through clinical trials. It is imperative that adolescents are given the opportunity to participate in relevant clinical trials that include novel therapies (Pappo, 2014).

## Methods

### Patient enrolment

We whole‐genome‐sequenced six patients as part of our study. Our first patient (M_4180), whose treatment we detail, was a 15‐year‐old female who attended the Department of Dermatology at the University Hospital Clínic of Barcelona, Spain. Five additional children with resected primary melanoma were also identified from the University Hospital Clínic of Barcelona and from Leiden University Medical Center, the Netherlands. The remaining cases were selected from a cohort of paediatric melanomas identified and sequenced at St Jude Children's Hospital, Memphis, TN (Lu et al., 2015), as part of the Paediatric Cancer Genome Project (Downing et al., 2012) study accession through the European Genome‐phenome Archive; EGAS00001000901 (Table S1). Written informed consent was obtained from the patients’ parents.

### Dermoscopy, histopathology and imaging

Total body photography and digital dermoscopy were performed by SP using MoleMax™ HD (Derma Medical Systems, Vienna, Austria) and DermLite^®^ FOTO (Dermlite^®^, San Juan Capistrano, CA, USA). Histopathological analyses were performed by an expert dermatopathologist.

### Sample processing

Tumour DNA extraction from the index 15‐year‐old patient (M_4180) was performed using the Qiagen DNA Micro Kit. Germline DNA was extracted from peripheral blood mononuclear cells using the salting out method.

### Tumour genomic analyses

DNA from a metastatic cutaneous deposit and whole blood DNA from the index 15‐year‐old patient were genome sequenced on the Illumina X10 platform (Table S2). Whole‐genome‐sequenced reads were aligned against the human reference genome (GRCh37) using the Burrows–Wheeler Aligner (Li and Durbin, 2009; Table S2). We used a somatic caller merging approach to identify somatic variants selecting only those detected using four or more algorithms for further analysis (Rashid et al., 2013). These calls were further filtered for germline polymorphic variants using the 1000 Genomes Project (Auton et al., 2015), and other standard quality filters were also applied (e.g. depth of coverage ≥10, read mapping quality ≥15). Small insertions and deletions were identified using Scalpel (Narzisi et al., 2014). Randomly selected candidate variants were validated by capillary sequencing. Large somatic copy number aberrations were detected using the Batternberg algorithm. Somatic variants from a series of 13 ‘conventional’ paediatric melanomas described by Lu et al. (2015) (so‐called due to their shared clinical and histological features typical of adult cutaneous melanoma) and for whom genome sequencing data were available were used for a comparative analysis. Exome sequencing data from a further 275 adult cutaneous melanomas were downloaded from The Cancer Genome Atlas and used for comparison with adult‐onset disease (Table S6).

### Germline genomic analyses

Germline DNA from the peripheral blood of five children with resected primary melanoma was whole‐genome‐sequenced on the Illumina HiSeq2500 platform (Tables S1 and S2). These sequences, and that of the index case, were combined with whole‐genome and whole‐exome sequences from a collection of 18 children sequenced at St Jude Children's Hospital (Lu et al., 2015) comprising 13 children from the ‘conventional’ melanoma cohort described above and five from the other histological subgroups described therein (Table S3). Germline variants were called using samtools mpileup (Li et al., 2009) and bcftools (Narasimhan et al., 2016). These variants were annotated for consequence using Ensembl Variant Effect Predictor (McLaren et al., 2016) and filtered for non‐synonymous variants. They were then further restricted to those variants known to be rare (allele frequency < 10^−3^) by comparison with the Exome Aggregation Consortium (ExAc) data set (Lek et al., 2016) or that were private to a single child.

### Data accession IDs

**Table:** 

M_4180_2728	EGAN00001232866	Tumour of patient M_4180
M_4180	EGAN00001195811	Germline of patient M_4180
M_509	EGAN00001197185	Germline of patient M_509
M_1064	EGAN00001197186	Germline of patient M_1064
M_3629	EGAN00001197187	Germline of patient M_3629
M_4117	EGAN00001197188	Germline of patient M_4117
D1_10_02707	EGAN00001197189	Germline of patient D1_10_02707

For details, see Table S1.

## Author contributions

Susana Puig identified the patient, provided the clinical history and undertook the dermoscopy as well as identifying four further suitable patients for this analysis. Susana Puig also secured informed consent. Remco van Doorn identified the patient from the University Hospital Leiden and secured informed consent. Marcelo Sánchez reviewed the radiology. Ana Arance and Carles Conill were the treating medical and radiation oncologists respectively. Joan Puig‐Butille was the clinical geneticist. Gemma Tell‐Marti extracted the tumour DNA for the patient described and Miriam Potrony collated the germline data from University Hospital Barcelona. Mamunur Rashid performed the bioinformatics analyses. Stefan Dentro undertook the copy number calls for this patient. Vivek Iyer analysed the germline sequencing data from the five further paediatric cases described, together with the germline data from the 18 children identified from Lu et al. (2015). Mamunur Rashid, Roy Rabbie and Carla Daniela Robles‐Espinoza critically analysed the somatic mutational data in the context of molecular data from The Cancer Genome Atlas. Roy Rabbie wrote the case history, prepared the clinical images and summarized the extended children's phenotypes. Roy Rabbie and Mamunur Rashid drafted the manuscript. Remco van Doorn, Pippa Corrie and Carla Daniela Robles‐Espinoza reviewed the manuscript and provided expert external dermatological, oncological and genomic input respectively. David Adams provided overall support and supervision including concept, analyses and critical manuscript revisions. All authors approved the final manuscript. The funders did not play a role in the design of this study or in the interpretation of the results.

## Funding

This work was supported by Cancer Research UK and the Wellcome Trust. The research at the Melanoma Unit in Barcelona is partially funded by Spanish Fondo de Investigaciones Sanitarias grants PI12/00840, PI15/00716 and PI15/00956; CIBER de Enfermedades Raras of the Instituto de Salud Carlos III, Spain, co‐financed by European Development Regional Fund ‘A way to achieve Europe’ ERDF; AGAUR 2014_SGR_603 of the Catalan Government, Spain; European Commission under the 6th Framework Programme, Contract No. LSHC‐CT‐2006‐018702 (GenoMEL) and by the European Commission under the 7th Framework Programme, Diagnoptics; a grant from ‘Fundació La Marató de TV3, 201331‐30’, Catalonia, Spain; a grant from Telemaraton of Spain ‘Todos somos raros’; and a grant from ‘Asociación Española Contra el Cáncer (AECC)’. The work was carried out at the Esther Koplowitz Center, Barcelona. MP is the recipient of a PhD fellowship FI14/00231 (PFIS) from Instituto de Salud Carlos III, Spain. RvD and NAG were supported by a grant from the Dutch Cancer Society (UL 2012–5489).

## Conflict of interest

No conflict of interests to declare.

## Supporting information


**Figure S1.** Cutaneous toxicities associated with vemurafenib in this patient.

**Figure S2.** Genome‐wide mutational landscape of the 15‐year‐old patient described displayed according to the 96 substitution classification described by Alexandrov et al. (2013).

**Table S1.** Clinical summary.
**Table S2.** QC metric of NGS data.
**Table S3.** Germline profile.
**Table S4.** All somatic mutations [M_4180].
**Table S5.** Exonic somatic mutations [M_4180].
**Table S6.** Mutation rate per megabase.
**Table S7.** All SNV validations.
**Table S8.** All CNVs from battenberg.
**Table S9.** All InDels from scalpel.
**Table S10.** Translocations from lumpy.
