# Supplementary figures and images for: Genomic analysis and clinical management of adolescent cutaneous melanoma
Source: Pigment Cell Melanoma Res. 2017 Apr 19;30(3):307–16. doi: 10.1111/pcmr.12574 (PMC5435926; doi:10.1111/pcmr.12574)

Supplementary Figure 1

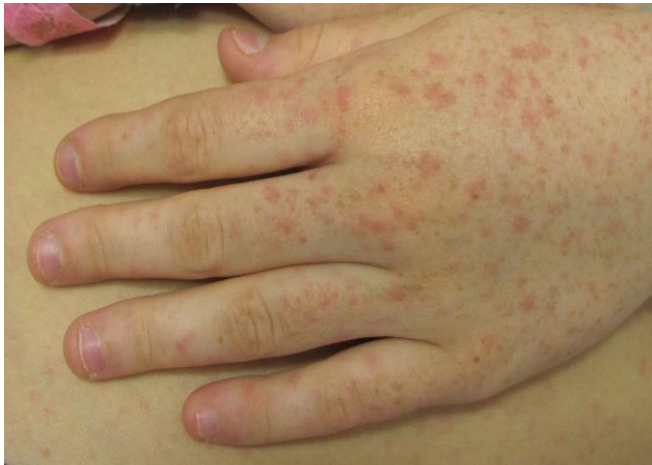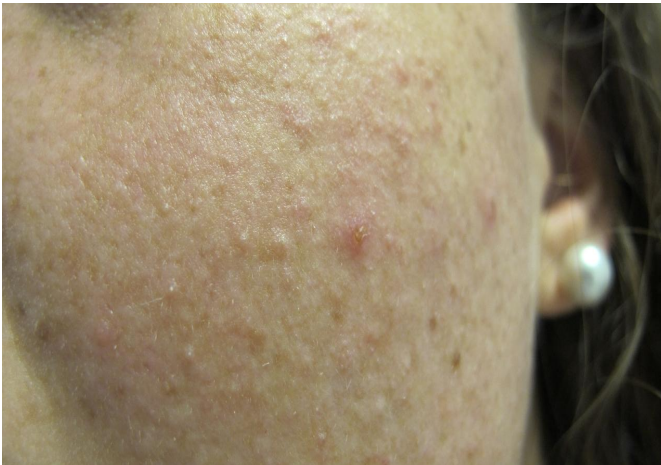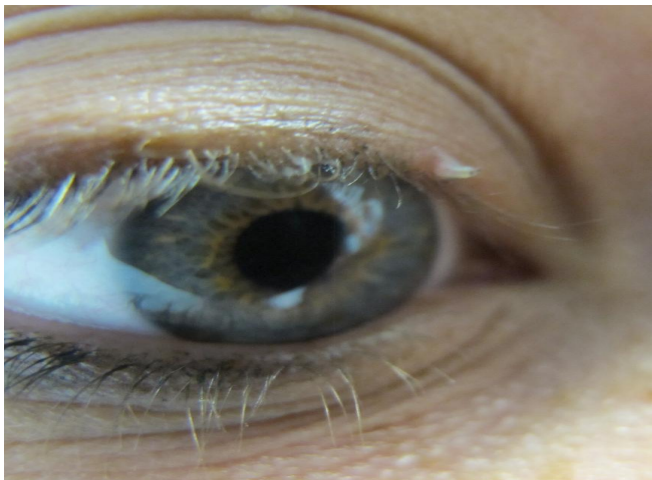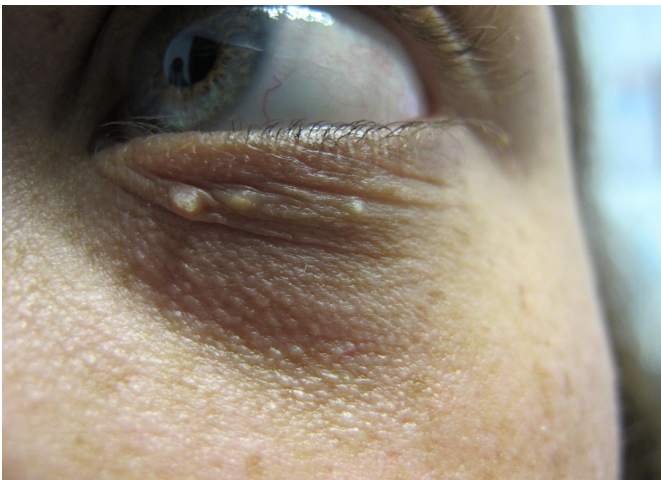

Supplement: Supplementary file 1 — Figure S1. Cutaneous toxicities associated with vemurafenib in this patient. [file PCMR-30-307-s001.pdf]

Supplementary Figure 2

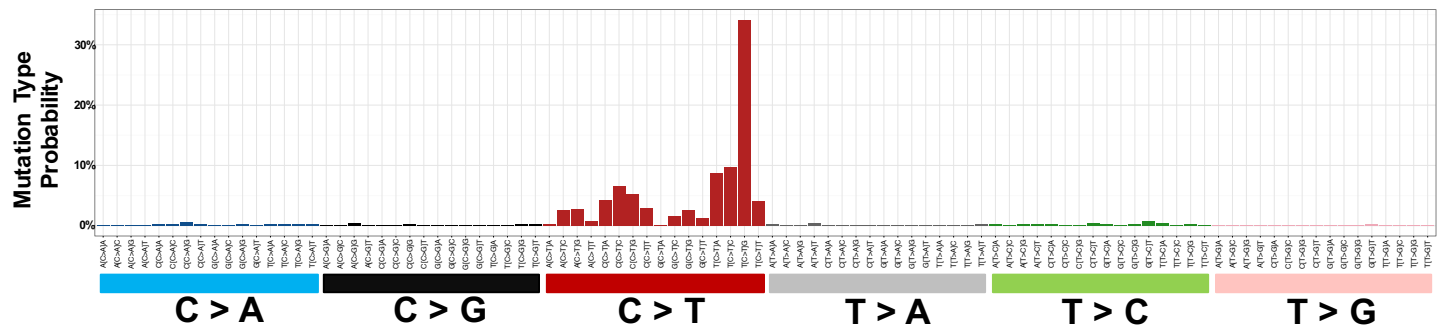

Supplement: Supplementary file 2 — Figure S2. Genome‐wide mutational landscape of the 15‐year‐old patient described displayed according to the 96 substitution classification described by Alexandrov et al. (2013). [file PCMR-30-307-s002.pdf]
